# Short-term culture of adult bovine ovarian tissues: chorioallantoic membrane (CAM) vs. traditional in vitro culture systems

**DOI:** 10.1186/s12958-018-0337-y

**Published:** 2018-03-09

**Authors:** Kylie Beck, Jaswant Singh, Mohammad Arshud Dar, Muhammad Anzar

**Affiliations:** 1 Agriculture and Agri-Food Canada, Saskatoon Research and Development Center, Canadian Animal Genetic Resource Program, S7N OX2, Saskatoon, SK Canada; 2 Departmnet of Veterinary Biomedical Sciences, Western College of Veterinary Medicine, University of Saskatchewan, Saskatoon, SK S7N 5B4 Canada; 3 Vaccination and Infectious Disease Organization, University Saskatchewan, Saskatoon, S7N 5E3 Canada

**Keywords:** Bovine, Ovary, In vitro culture, Chick chorioallantoic membrane, Angiogenesis, Follicle

## Abstract

**Background:**

A suitable culture system is important for follicle growth in adult bovine ovarian tissue. This study aimed to assess the avian chorioallantoic membrane (CAM) for short-term culture of adult bovine ovarian tissues compared with a traditional in vitro culture system.

**Methods:**

Ovarian cortical tissues (1–2 mm^3^), collected from slaughtered adult cows, were randomly assigned to control, CAM or in vitro culture groups. In the control group, ovarian tissues were fixed with paraformaldehyde without culture. In CAM and in vitro culture groups, the ovarian tissues were cultured for up to 5 days and then fixed. Ovarian tissues were examined on culture days 0, 1, 3 and 5 for angiogenesis, follicle morphology and growth. In all groups, primordial and growing (healthy and atretic) follicle densities were determined.

**Results:**

In the CAM culture, the avian blood vessel density increased (*p* < 0.01) over time with a decline (*p* < 0.001) in the bovine blood vessel density. Healthy primordial, atretic primordial and healthy growing follicle densities were higher (*p* < 0.05) in CAM-cultured ovarian tissues than in vitro-cultured tissues. Regardless of the culture system, the density of healthy primordial follicles decreased (*p* < 0.001) over time with an increase in healthy growing follicles on day 3 (*p* < 0.01) and an increase in atretic (primordial and growing) follicles during the 5-day culture period (*p* < 0.001). The proportions of healthy primordial and atretic growing follicles were also affected by culture day (*p* < 0.001).

**Conclusions:**

The CAM culture in chick embryos supported the bovine ovarian tissue grafts for 3 days demonstrating that CAM can be used as a satisfactory short-term culture system to assess ovarian tissue health, and to study follicle activation and development.

## Background

Animal cells and tissues require an efficient culture system that closely mimics their “natural” conditions when cultured in vitro. In vitro culture provides nutrients and energy to cells and tissues for their development and growth. The organ-culture of intact ovaries from newborn mice for 8 days followed by in vitro culture of retrieved oocyte-granulosa cell complexes for an additional 14 days, led to the birth of healthy pups following in vitro fertilization and embryo transfer [1]. In vitro culture of whole mouse ovaries in fibrin-alginate hydrogel matrix also yielded mature oocytes competent to produce embryos in vitro [2]. Furthermore, mouse ovarian tissues xenografted onto immunodeficient nude rats produced mature oocytes which were fertilized and developed into fertile adult mice [3]. In vitro culture requires a CO_2_ incubator with high humidity and media; whereas, tissue xenografting requires immunodeficient mice, surgical skills and animal care. The chorioallantoic membrane (CAM) of chick embryo is an intermediate culture system between simple in vitro and complex in vivo xenografting culture systems. Human and fetal bovine ovarian tissues have been previously cultured in CAM [4–6]. Other cell types have been shown to develop in the CAM culture system suggesting its usefulness in regenerative medicine [7]. The CAM culture system is a popular model to study the acute toxicology of anti-cancer agents [8] and to investigate angiogenesis in human ovarian cancer [9]; however, little information is available on the use of CAM culture for adult bovine ovarian tissues.

The avian CAM is a multilayered structure lined by the ectodermal epithelium at the interface with air, mesoderm (or stroma) containing the blood vessels and the endoderm at the interface with the allantoic sac [10]. The extracellular matrix of CAM is similar to that of the peritoneum in mammals, which is a common site for orthotopic autotransplantation of ovarian tissue [11]. Chick membranes form a bursal-like structure around fetal bovine ovarian tissue and create a microenvironment similar to that seen in vivo [5]. Follicle development has been studied in human [4, 11, 12], mouse [6] and cat [13] ovarian tissues after CAM culture for 5–10 days. The CAM culture of fetal bovine ovarian tissues for even 10 days did not activate primordial follicles [5, 6].

In the Canadian Animal Genetic Resource (CAGR) program, efforts have been focused on conserving the genetic diversity of Canadian livestock species. To achieve this goal, semen, embryos, testis and ovaries are collected, especially from endangered species, for conservation purposes. Keeping in view the scattered livestock population in Canada’s extensively large geographical area, the transport of ovaries to the CAGR program under field conditions is quite challenging. Therefore, there is a dire need to develop a suitable short-term culture system that can be mobile and maintain tissue viability during transport under field conditions. So far, there are no reports on the use of the CAM culture system with adult bovine ovarian tissue. This study was designed to test the hypothesis that CAM culture can maintain the health of adult bovine ovarian tissue for a short period. The specific objectives of this study were to study avian angiogenesis in adult bovine ovarian tissues cultured in CAM, and to compare the follicle development between CAM and in vitro culture systems.

## Methods

Ovaries were collected from adult beef cows (> 5 years old) killed at a slaughterhouse located 130 km away from the laboratory. Ovaries were held at 4 °C and brought to the laboratory within six hours. After washing (2×) in cold (4 °C) sterile 0.9% saline, ovaries (*n* = 5) without a corpus luteum were selected and cortical slices (1–2 mm^3^) were cut in cold (4 °C) Dulbecco’s phosphate buffer saline (DPBS). From each ovary, 3 slices were randomly assigned to the control group (untreated, no culture), and 9 slices to each of CAM and in vitro culture group, as described below. This experiment was repeated 5 times over a 2-month period (i.e. 1 ovary per day). All chemicals were purchased from Sigma-Aldrich, St. Louis, MO, USA, unless otherwise mentioned.

### Control group

Ovarian cortical slices were fixed with 4% (*w*/*v*) paraformaldehyde immediately after dissection (culture day 0) to provide a baseline comparison of the development of primordial and growing (healthy and atretic) follicles over the 5-day culture period.

### CAM culture group

Fertilized eggs were procured from the University of Saskatchewan Poultry Centre and brought to the laboratory. Eggs were gently wiped with 70% (*v*/v) ethanol and incubated at 37 °C and 62% relative humidity in an egg incubator (1502 Digital Sportsman Model; GQF Manufacturing Company, Savannah, GA, USA) equipped with moving shelves to turn the eggs. The chorioallantoic membrane of the chick embryo was exposed and processed as described earlier [11], with some modifications. Briefly, on embryonic day 3 of incubation, the eggs were candled to locate the embryonic disc. A window (approximately 1 × 2 cm) was made in the eggshell with surgical scissors. To accommodate ovarian tissue, 2 ml albumin was aspirated from each egg with an 18-gauge needle, fitted on a 5-mL syringe, directed towards the apex of the egg to avoid damage to the embryo. A piece of clear tape was placed over the window to prevent dehydration, and the eggs were placed back in the incubator with the window facing upward. Embryos were visually checked every 48 h for embryonic movement and heartbeat, and CAM blood vessel movement.

On embryonic day 10 (culture day 0), a small area of CAM was gently traumatized by touching it briefly (< 1 s) with a sterilized piece of lens paper (4–5 mm^2^) dipped in sterile acetone. This procedure removed the top epithelial layer, exposing the underlying blood vessels while keeping the basal layer intact. Two slices from each bovine ovary were directly plunged into liquid nitrogen without cryoprotectants and then thawed in DPBS (3×). These were designated dead tissues to serve as negative controls for angiogenesis. Bovine ovarian cortical slices (9 live and 2 dead per ovary) were gently placed in the CAM using sterile microsurgical forceps (1 slice per egg). Live and dead tissue slices were grossly examined for angiogenesis characterized by the movement of avian blood vessels towards the graft on days 1, 3 and 5 of CAM culture. Three live tissue slices were removed from the CAM on days 1, 3 and 5, whereas two dead tissue slices were removed on day 5 for histological examination. The embryos were killed by placing them in a − 20 °C freezer, as approved by the Animal Care Committee, University of Saskatchewan (Animal Use Protocol # 20130068).

#### In vitro culture group

Ovarian tissue slices were placed on tissue culture inserts (9 slices/ovary, 3 slices/insert; 0.4 μm pore size; Millicell-CM, EMD Millipore Corp., Billerica, MA, USA) in 6-well plates containing 1.2 mL of culture medium composed of TCM199 supplemented with 1% (*v*/v) Gibco® Insulin-Transferrin-Selenium supplement (100×; 1 g/L insulin, 0.55 g/L transferrin, and 0.00067 g/L selenium; Thermo-Fisher Scientific, Nepean, ON, Canada), 100 mIU/mL recombinant follicle stimulating hormone (rFSH or Gonal-f; Serono, Switzerland), 100 μg/mL penicillin, and 100 μg/mL streptomycin. The ovarian tissues were cultured at 37 °C in 5% CO_2_ within a humidified incubator (Forma Scientific Inc. Marietta, OH, USA). Three tissue slices from 1 culture insert were removed for histological examination on days 1, 3 and 5.

### Histological examination

For histological examination, CAM and in vitro cultured ovarian tissues were fixed with 4% (*w*/*v*) paraformaldehyde, embedded in paraffin wax, serially sectioned at 5 μm thickness, stained with hematoxylin and eosin, and examined under a light microscope (Axioskop 40, Carl Zeiss Canada Ltd., North York, ON, Canada). From each tissue block, 8 serial sections were placed on each glass slide followed by 8 more serial sections after discarding 10 intervening sections (a total of 50 μm between adjacent slides). Slides were examined blindly for primordial or growing (healthy or atretic) follicles. To avoid recounting, a follicle with a visible nucleolus was counted only once in the section. The first section on each slide acted as a reference section and was not counted; thus, a total thickness of 280 μm (7 sections of 5 μm thickness per slide × 8 slides) spanning the majority of the ovarian fragment (8 slides of 35 μm counting thickness + 7 inter-slide gaps of 55 μm = 665 μm) was counted. Each ovarian section was observed at 40× magnification and the area of the section and follicle numbers were determined using FIJI / ImageJ software [14]. For photomicrography, the ovarian sections were imaged at 10× and 40×.

Morphological classification of follicles was based on the definitions previously described [15], with slight modifications. Each follicle was described as a primordial follicle (containing an oocyte surrounded by a single layer of flattened granulosa cells) or a growing follicle (containing an oocyte surrounded by one or more layers of cuboidal granulosa cells). Follicles were classified as healthy (spherical in shape, homogeneous ooplasm, slightly granulated nucleus with condensed chromatin, and even distribution of granulosa cell layers) or atretic (misshapen oocyte with or without vacuoles, and partially or fully disrupted granulosa cell layer with pyknotic nuclei). Avian- and bovine-origin blood vessels were recorded based on the presence or absence of nucleated red blood cells, respectively.

### Stereology

The area and volume of ovarian tissues were estimated by standard stereological analysis using FIJI / ImageJ software v1.49 [14]. To determine the area of each ovarian section, the “grid” plug-in of FIJI software was used. An overlay grid of evenly spaced crosses (at 100 μm distance from each other in the x and y axis) was placed randomly on the image and the crosses overlapping the image were counted. Each cross represented 10,000 μm^2^ of area and the total number of squares was multiplied by the thickness of the section (5 μm) to determine the tissue volume per section. The sum of volumes of all counted sections determined the total tissue volume examined for each ovary. Follicle and blood vessel densities were calculated by dividing the number of follicles or blood vessels detected in all counted sections by the total volume of each respective tissue fragment.

### Statistical analysis

Follicle and blood vessel densities were analyzed by analysis of variance and a 2 (culture types) × 4 (culture days) repeated measures factorial design using SAS® Enterprise Guide 4.2. The normality of the residuals was evaluated before final analysis and if the values did not meet the criterion data were transformed using log transformation. Endpoints were summarized by mean ± standard error of the mean (SEM) for each group. Replicate number (ID; 1–5), day in culture (0, 1, 3 or 5), culture type (1 = in vitro, 2 = CAM) and follicle densities were tabulated for each ovarian tissue group. The syntax of the SAS program included: Proc mixed covtest; class ID day culture density; model density = time culture time*culture / DDFM = kr htype = 3; repeated culture (day) /subject = ID type =??; lsmeans time / pdiff adjust = tukey; lsmeans culture / pdiff adjust = tukey; lsmeans time*culture / pdiff adjust = tukey; run. Eleven covariate matrices (variance components, compound symmetry, heterogenous compound symmetry, toeplitz, banded-toeplitz, huynh-feldt, autoregressive, heterogenous autoregressive, ante-dependence, unstructured and banded-unstructured) were initially tested (by replacing the “??” in the above syntax with the covariate code) to select the optimal model type based on the smallest AICC value from the mixed procedure program. The level of statistical significance was set at *p* < 0.05. If the main effects or interaction term had *p* ≤ 0.05, for the selected model, post-hoc comparisons were conducted using Tukey’s adjustment.

## Results

### Embryo survival

In the CAM culture group, total 121 eggs were used. The embryo survival rates were 89% (109/121) from windowing (embryonic day 3) to ovarian tissue grafting (embryonic day 10) and 99% (108/109) from grafting (embryonic day 10) to ovarian tissue retrieval (embryonic day 15).

### Angiogenesis

**Fig. 1:**
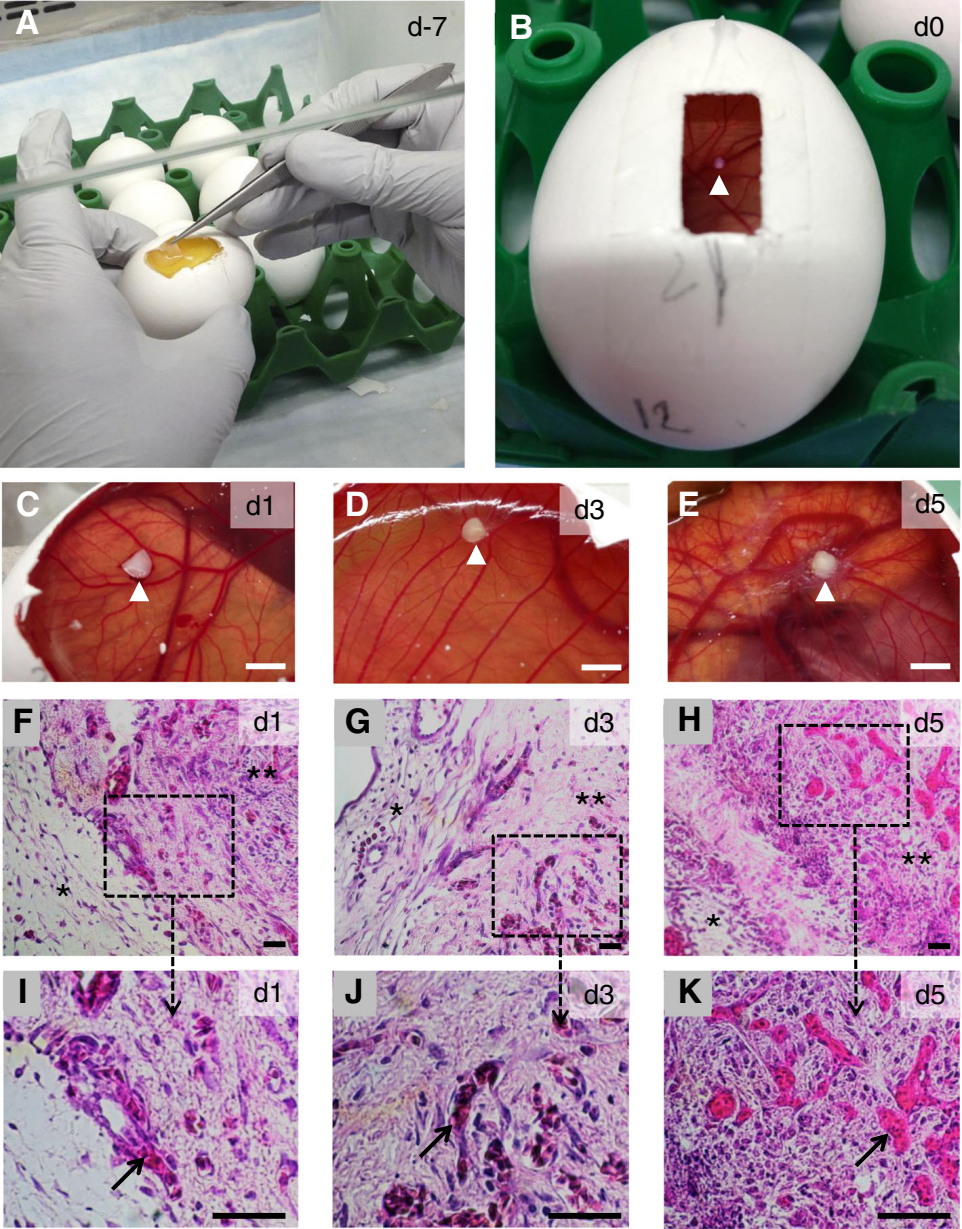
Angiogenesis in bovine ovarian tissue cultured on traumatized chorioallantoic membrane (CAM) of chick embryos. **a** Windowing (1 × 2 cm) in eggshell on embryonic day (d) 3 (7 days before culture). **b** Grafting of bovine ovarian tissue (white arrowhead) on embryonic day 10 (culture day 0). **c-e** Blood vessel movement (pinwheel-like) around the ovarian tissue on culture days 1, 3 and 5. **f-h** Histological examination of the ovarian tissue on culture days 1, 3 and 5. Asterisk (*) and double asterisk (**) represent CAM tissue and bovine ovarian tissue respectively. **i-k** Higher magnification of dotted areas in panels F-H. Black solid arrows indicate avian blood vessels characterized by nucleated red blood cells in bovine ovarian tissues. White bars (**c-e**) and black bars (**f-k**) represent 2 mm and 50 μm, respectively

**Fig. 2:**
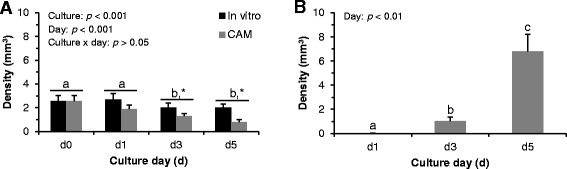
Effects of culture systems, culture days (d) and their interaction on bovine blood vessel density (**a**), and the effect of CAM culture day (d) on avian blood vessel density (**b**). Each bar represents mean ± SEM (*N* = 5 ovaries; 3 slices/ovary/group/day). Bovine blood vessel density varied significantly by culture systems and culture days; whereas, avian blood vessel density influenced significantly by culture days. Within a blood vessel type, bars with different superscripts (a,b,c) represent significant difference between culture days (*p* < 0.05). An asterisk (*) represents significant difference between culture systems (*p* < 0.05), on a specific day

A window in an eggshell and grafted ovarian tissue are illustrated in Fig. 1a and b. Gross evaluation revealed a pinwheel-like movement of small avian blood vessels towards ovarian tissue in CAM cultures by day 3, which was further established by day 5 (Fig. 1c-e). There was no apparent change in the size of ovarian tissue during CAM incubation. Histological evaluation demonstrated the proximity between the CAM and bovine ovarian tissues as well as infiltration of avian blood vessels into bovine ovarian tissues (Fig. 1f-k). The overall density of bovine blood vessels in ovarian tissue was higher (*p* < 0.001) in in vitro cultures compared to the CAM cultures. However, density declined (*p* < 0.05) on days 3 and 5 compared with days 0 and 1 (Fig. 2). The density of avian blood vessels in the bovine ovarian tissue cultured in CAM increased from 1 to 7 blood vessels/mm^3^ between days 3 and 5 (*p* < 0.05; Fig. 2). After the 5-day CAM culture period, there was negligible movement of avian blood vessels towards dead-ovarian tissue (negative control) on gross examination and lack of avian blood vessel infiltration on histological examination (data not shown).

### Follicle morphology and growth

**Fig. 3:**
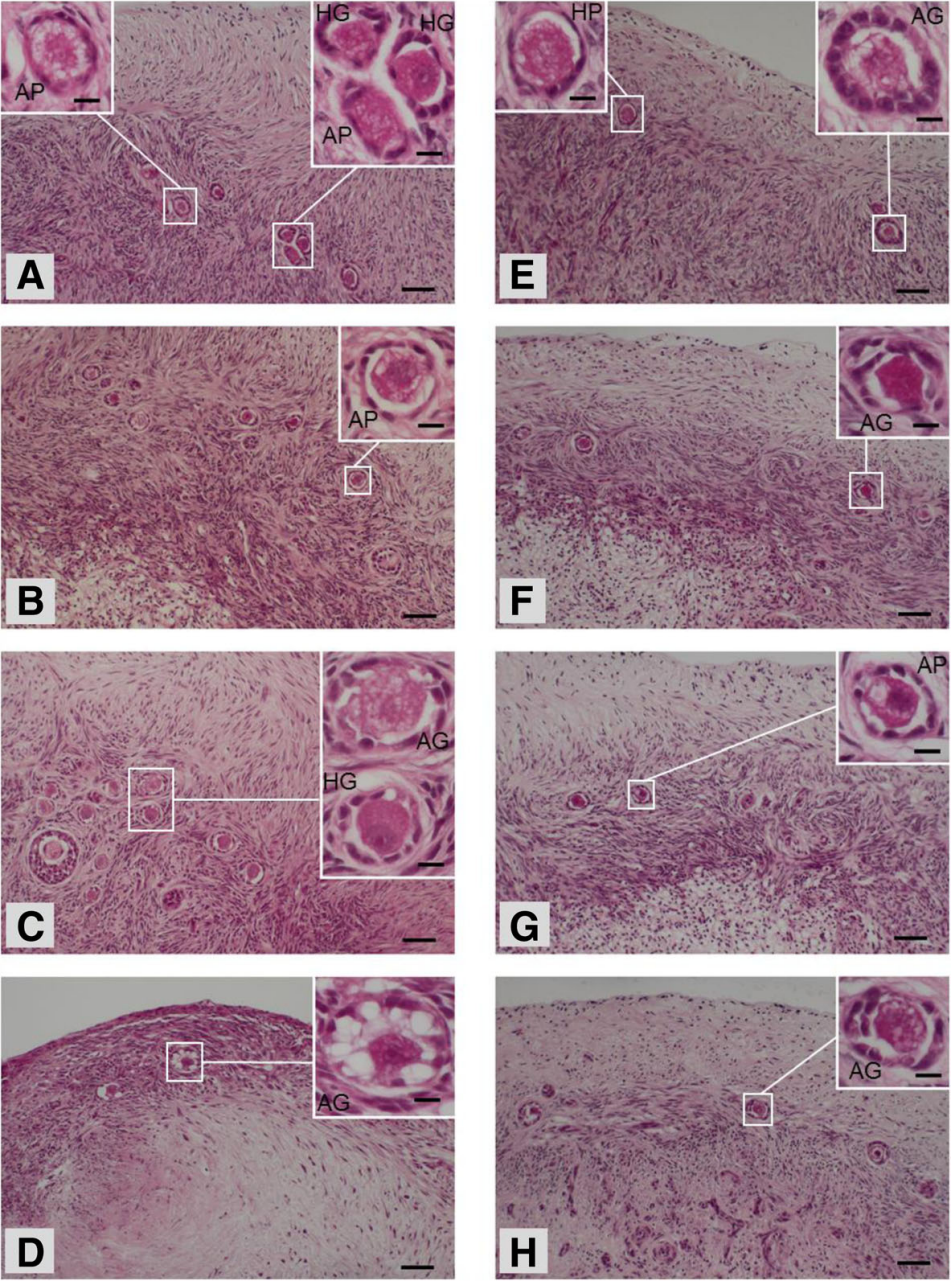
Hematoxylin and eosin staining of bovine ovarian tissues from chorioallantoic membrane (CAM) and in vitro cultures on day (d) 0, 1, 3 and 5. **a** Histological section of CAM cultured bovine ovarian tissue on day 0 showing several follicles. Upper right insert represents an enlarged image of two healthy growing [HG] and one atretic primordial [AP] follicle. Upper left insert represents an enlarged image of an atretic primordial [AP] follicle. Scale bar = 50 μm. Insert scale bar = 10 μm. **b** Histological section of CAM cultured bovine ovarian tissue on day 1 showing several follicles. Upper right insert represents an enlarged image of an atretic primordial [AP] follicle. Scale bar = 50 μm. Insert scale bar = 10 μm. **c** Histological section of CAM cultured bovine ovarian tissue on day 3 showing several follicles. Upper right insert represents an enlarged image of healthy growing [HG] and atretic growing [AG] follicles. Scale bar = 50 μm. Insert scale bar = 10 μm. **d** Histological section of CAM cultured bovine ovarian tissue on day 5 showing two follicles. Upper right insert represents an enlarged image of an atretic growing [AG] follicle. Scale bar = 50 μm. Insert scale bar = 10 μm. **e** Histological section of in vitro cultured bovine ovarian tissue on day 0 showing few follicles. Upper right insert represents an enlarged image of an atretic growing AG] follicle. Upper left insert represents an enlarged image of a healthy primordial [HP] follicle. Scale bar = 50 μm. Insert scale bar = 10 μm. **f** Histological section of in vitro cultured bovine ovarian tissue on day 1 showing few follicles. Upper right insert represents an enlarged image of an atretic growing [AG] follicle. Scale bar = 50 μm. Insert scale bar = 10 μm. **g** Histological section of in vitro cultured bovine ovarian tissue on day 3 showing few follicles. Upper right insert represents an enlarged image of an atretic primordial [AP] follicle. Scale bar = 50 μm. Insert scale bar = 10 μm. **h** Histological section of in vitro cultured bovine ovarian tissue on day 5 showing few follicles. Upper right insert represents an enlarged image of an atretic growing [AG] follicle. Scale bar = 50 μm. Insert scale bar = 10 μm

**Fig. 4:**
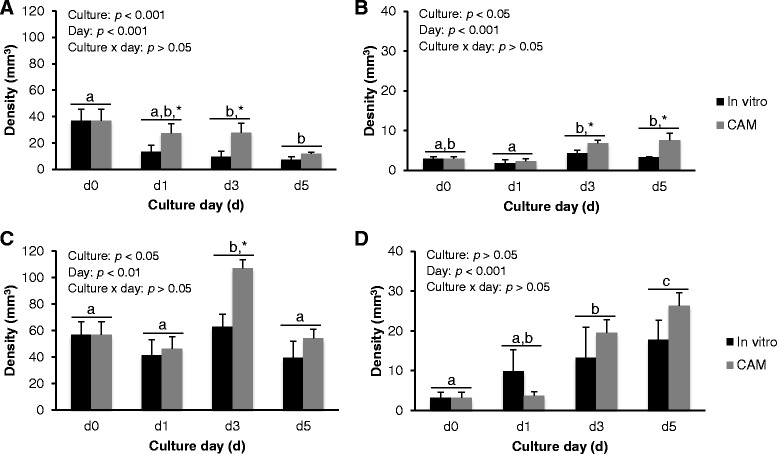
Effects of culture systems, culture days (d) and their interaction on (**a**) healthy primordial [HP]; **b** atretic primordial [AP]; **c** healthy growing [HG]; and (**d**) atretic growing [AG] follicle densities. Each bar represents mean ± SEM (*N* = 5 ovaries; 3 slices/ovary/group/day). Healthy primordial, atretic primordial and healthy growing follicle densities were affected significantly by culture systems and culture days; whereas, atretic growing follicle density was influenced significantly by culture days only. Within a follicle population, bars with different superscripts (a, b, c) represent significant difference between culture days (*p* < 0.05). An asterisk (*) represents significant difference between culture systems (*p* < 0.05), on a specific day

Both types of bovine ovarian tissues (CAM and in vitro cultures) appeared similar on days 0, 1, 3 and 5 upon histological examination (Fig. 3). Primordial and growing (healthy and atretic) follicles were randomly distributed in the CAM and in vitro culture groups and follicle densities in CAM- and in vitro-cultured ovarian tissues on days 0, 1, 3 and 5 are presented in Fig. 4. Healthy primordial, atretic primordial and healthy growing follicle densities were affected significantly by culture system and culture day. The atretic growing follicles were influenced significantly by culture day only (Fig. 4). The culture system x culture day interaction was not significant in all follicle populations. Therefore, data on follicle densities were pooled within culture system or culture day. Healthy primordial, atretic primordial and healthy growing follicle densities were higher (*p* < 0.05) in CAM cultures on days 1 and 3, days 3 and 5, and day 3 respectively, compared to in vitro cultures. During the incubation period, the density of healthy primordial follicles declined (*p* < 0.05) on culture days 3 and 5 compared to day 0, with a corresponding increase (*p* < 0.05) in the atretic primordial follicles on day 3, healthy growing follicles on day 3, and atretic growing follicles on days 3 and 5.

**Fig. 5:**
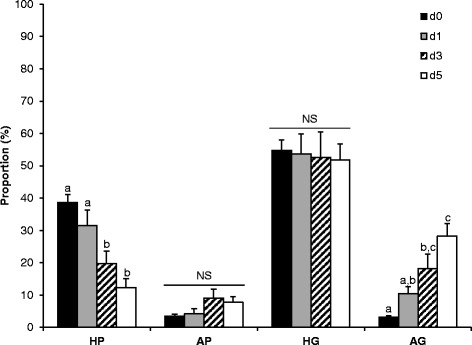
Effect of culture days (d) on proportions of healthy primordial [HP], atretic primordial [AP], healthy growing [HG] and atretic growing [AG] follicles, averaged over culture system. Each bar represents mean ± SEM (N = 5 ovaries; 3 slices/ovary/group/day). Proportions of healthy primordial and atretic growing follicles were affected significantly by culture days (*p* < 0.05). Within a follicle type, bars with different superscripts (a, b, c) represent significant difference between culture days (*p* < 0.05)

The proportion of primordial and growing (healthy and atretic) follicles was not affected (*p* > 0.05) by the culture system. Therefore, data were pooled within culture systems. The proportions of healthy primordial and atretic growing follicles differed by culture day (*p* < 0.001). The proportion of healthy primordial follicles was the highest on culture day 0 and declined on day 3 (*p* < 0.05; Fig. 5). Correspondingly, the proportion of atretic growing follicles was the lowest on culture day 0 and increased (*p* < 0.05) linearly during the 5-day culture period (Fig. 5). The proportions of atretic primordial and healthy growing follicles did not differ by culture day (*p* > 0.05).

## Discussion

Based on angiogenesis and follicle development, CAM was found to be another satisfactory short-term culture system for adult bovine ovarian tissues. The infiltration of avian blood vessels indicated that the CAM culture system supported bovine ovarian tissues. Healthy primordial and growing follicles were found in both CAM and in vitro culture systems. The healthy primordial follicle density declined over the 5-day culture period with an increase in healthy growing follicles, and atretic primordial and growing follicles.

The chorioallantoic membrane of chick embryos has extra-embryonic blood vessels and is not innervated, thus it provides a suitable transplantation site for a variety of tissues. In this study, the high survival rate (> 90%) of chick embryos indicated their tolerance to adult bovine ovarian tissues. It is noteworthy that adult bovine ovarian tissues were grafted on embryonic day 10 and removed on embryonic day 15; whereas, the chick embryo becomes fully immunocompetent on embryonic day 18 [16]. Similar survival rates of chick embryos have been reported after grafting of human ovarian tissues [4, 11].

In this study, a pinwheel-like movement of avian blood vessels around bovine ovarian tissues was noticed on culture day 3, which became more prominent on day 5. A radial arrangement of blood vessels converging towards the graft considered to be an evidence of angiogenesis [7]. Blood supply is important for primordial and early growing follicles in the ovarian cortex which is devoid of vasculature and follicles rely on the neighboring blood vessels [17]. Following autotransplantation, the rapid angiogenesis around bovine ovarian tissue restored ovarian function with minimum follicle loss due to ischemia [18]. The “living” ovarian tissue produced angiogenic factors which facilitated the movement of endothelial cells towards the ovarian graft [19]. In this study, the ovaries were collected from slaughtered cattle; therefore, a decline in the density of bovine blood vessels in ovarian tissues over time was obvious and expected in both culture system. In the CAM culture, there was a significant increase in the density of avian blood vessels between day 3 and day 5. This indicated that ovarian tissues remained healthy during 5-day CAM culture since avian blood vessels did not infiltrate into the dead ovarian tissues (negative control). Although infiltration of avian blood vessels has been reported in fetal bovine [5, 6], adult domestic cat [13] and adult human [4, 11] ovarian tissues during CAM culture, the follicle growth response varied by species. In this study, only cortical ovarian tissues, possessing minor blood vessels, were cultured in CAM. Previously, the CAM culture of human ovarian cortical tissues along with medulla improved angiogenesis without any beneficial effect on follicle growth [4]. In this study, avian and bovine red blood cells were differentiated based on the presence and absence of a nucleus, respectively, using hematoxylin and eosin staining. In the human-mouse model, red blood cells of both species are anucleated and the origin of blood vessels was detected using anti-von Willebrand factor (anti-vWF) and anti-mouse CD31 [20] or desmin [4] immunostaining, a laborious and expensive technique compared to hematoxylin and eosin staining.

In this study, there were no marked histological differences in ovarian tissues from CAM and in vitro cultures. Healthy primordial and growing follicles were found in both CAM and in vitro culture systems. The CAM culture system yielded a higher density of healthy primordial follicles on culture days 1 and 3, atretic primordial follicles on days 3 and 5, and healthy growing follicles on day 3 than the in vitro culture system. In CAM culture, the development of healthy growing follicles from 57/mm^3^ (culture day 0) to 107/mm^3^ (culture day 3) confirmed that CAM culture supported bovine ovarian tissues. This study indicated that the CAM culture yielded higher healthy growing follicles on day 3 than in vitro culture of adult bovine ovarian tissue. Follicle growth was similar between CAM and in vitro cultures in human ovarian tissues [4] while in vitro culture was superior to CAM in domestic cat ovarian tissue [13].

Healthy primordial follicles are destined to undergo either activation leading to growing follicles, or atresia. In this study, the density of healthy primordial follicles decreased and densities of healthy growing and atretic (primordial and growing) follicles increased. A decrease in the density of primordial follicles with an increase in the density of growing follicles during CAM culture was attributed to follicle activation, as reported in fetal bovine ovarian tissues [21]. In this study, the density of healthy growing follicles reached a maximum on day 3, which was synchronous with the initiation of angiogenesis around and inside ovarian tissue. A decline in the density of healthy growing follicles between day 3 and day 5 and corresponding increase in atretic growing follicles may be attributed to insufficient avian vascularization for follicle sustenance. Following the xenotransplantation of ovarian tissue, the loss of follicles is mainly due to ischemia and hypoxia until revascularization is established [22, 23]. The CAM culture of fetal bovine ovarian tissues as long as for 10 days did not activate the primordial follicles to primary follicles whereas follicle activation occurred on day 2 in in vitro culture. In the same study, the CAM culture did not support the primary follicles previously activated in vitro, but ovarian tissues cultured in CAM first and transferred to in vitro culture resumed follicle activation [5]. Later, fetal bovine ovarian tissues cultured in CAM of gonadectomized chick embryo showed the activation of primordial follicles to primary follicles [6]. Anti-Müllerian hormone (AMH), naturally occurring in chick embryos, has been shown to have an inhibitory effect on follicle activation in fetal bovine and newborn murine ovarian tissue [5, 6]. Perhaps the size of the cortical pieces used in this study were large enough to slow down the vascularization, so that follicles were able to activate before the avian AMH (~ 140,000 Da) penetrated the graft either by diffusion or in the new blood vessels. Moreover, an increase in the density of healthy growing follicles on culture day 3 is in agreement with previous findings that AMH initiated follicular growth in adult human and rat ovarian tissues [24, 25]. Thus, AMH can inhibit or stimulate follicle activation in ovarian tissue depending upon species and age of animal (fetal vs. adult) [11].

In the present study, the proportion of healthy primordial follicles declined with an increase in atretic growing follicles whereas the proportions of atretic primordial and healthy growing follicles did not change during the incubation period. It is anticipated that primordial follicles are activated into growing follicles while older growing follicles undergo atresia or degeneration during incubation. Our results showed that the morphological status (healthy or atretic) of growing follicles was independent of the culture system. This is in agreement with earlier findings on human ovaries [4] and fetal bovine ovaries [5]. Frozen-thawed human cortical tissues cultured in CAM for 5 days yielded 87% healthy follicles (primordial, primary and secondary) [4]. In the present study, the unfrozen bovine cortical tissues yielded 64% healthy follicles (12% primordial and 52% growing). This difference may be due to species and reproductive health of donor females. Previously, vaccines, hormones and nutrients have been successfully injected into the air cell, albumin, amniotic fluid, allantoic fluid and yolk sac of chick embryos on different days of incubation [26–28]. It would be quite interesting to assess whether follicle stimulating hormone (FSH) injection of chick embryos improves the growth of activated follicles in ovarian tissues. Furthermore, in the present study, the proportion of healthy growing follicles was higher than that of healthy primordial follicles (55% vs. 40%), although the reason for this is unknown. We are confident in the follicle evaluation criteria used in this study. Unfortunately, the reproductive history including age, parity, lactation, disease and fertility status of culled animals was not available. Moreover, the estimation of ovarian follicle densities in human ovarian biopsies was extremely variable due to the uneven distribution of follicles over the surface of ovary [29].

The majority of follicles (~ 95%) were healthy (primordial and growing) and the remaining follicles were atretic. Atresia is the most common outcome for ovarian follicles and may occur at any stage of the estrous cycle [30]. There is a possibility that follicle degeneration and atresia occurred during the transport of ovaries from the slaughterhouse. Bovine ovaries are commonly procured from local slaughterhouses and transported to the laboratory at > 30 °C [31]. In the present study, the slaughterhouse was located quite far from our laboratory. Therefore, ovaries were kept on ice during transit to minimize autolysis.

The CAM culture system may be helpful for transport of ovarian tissues collected from endangered species, for conservation purposes. Moreover, the CAM culture system can be used to assess the health of tissue following any physical or chemical shock. The CAM culture was found to be a suitable model to develop a cryopreservation protocol for human ovarian tissue [4]. In North America, slaughterhouses operate under strict regulations of federal and provincial (state) governments. Therefore, the use of ovarian tissues from slaughtered animals does not pose tough ethical restrictions.

Like other techniques, the CAM culture system has few disadvantages and limitations. Special training is required on eggshell windowing and chorioallantoic membrane preparation for ovarian tissue grafting. The sacrifice of a chick embryo for 3-day incubation is questionable. The chick embryo has to remain alive throughout the incubation period to support the ovarian tissue. The development of primordial follicles to secondary or tertiary follicles requires longer than 3 days. So far, no attempt at long-term CAM culture by removing ovarian tissue from one CAM and re-grafting it onto another fresh CAM, has been made. The CAM system in human assisted reproductive technologies requires special permission to address associated ethical issues. The use of CAM culture for human fertility preservation should be considered with great caution due to the risk of infection from hen’s eggs [3, 11].

## Conclusions

The CAM culture system supported adult bovine ovarian tissues. The ovarian tissues cultured in CAM underwent angiogenesis and avian blood vessel infiltration which indicated the healthy ovarian tissues. The higher densities of healthy growing follicles on day 3 of CAM culture demonstrated that CAM can be used as a satisfactory short-term culture system for the adult bovine ovarian tissue.

## Abbreviations

CAM: Chorioallantoic membrane
TCM: Tissue culture medium
